# Test–retest reliability of peripheral arterial tonometry in the metabolic syndrome

**DOI:** 10.1177/1479164114525971

**Published:** 2014-03-21

**Authors:** Katherine A Sauder, Sheila G West, Cindy E McCrea, Janice M Campbell, Alexandra L Jenkins, David JA Jenkins, Cyril WC Kendall

**Affiliations:** 1 Department of Biobehavioral Health, The Pennsylvania State University, University Park, PA, USA; 2 Department of Nutritional Sciences, The Pennsylvania State University, University Park, PA, USA; 3 Glycemic Index Laboratories Inc., Toronto, ON, Canada; 4 Clinical Nutrition and Risk Factor Modification Center, St. Michael's Hospital, Toronto, ON, Canada; 5 Department of Nutritional Sciences, University of Toronto, Toronto, ON, Canada; 6 Department of Medicine, Division of Endocrinology and Metabolism, St Michael's Hospital, Toronto, ON, Canada; 7 College of Pharmacy and Nutrition, University of Saskatchewan, Saskatoon, Saskatchewan, Canada

**Keywords:** Peripheral arterial tonometry, endothelial function, reliability, metabolic syndrome

## Abstract

Endothelial dysfunction is an important contributor to atherosclerosis and cardiovascular disease. However, routine assessment via angiography or flow-mediated dilation is difficult due to technical limitations. Peripheral arterial tonometry (PAT) is a promising alternative method for non-invasive assessment of endothelial dysfunction. This study assessed the test–retest reliability of PAT in adults with the metabolic syndrome (n = 20) and provides sample size and power estimates for study design. Participants completed five PAT tests each separated by 1 week. The PAT-derived reactive hyperaemia index (RHI) showed robust repeatability (intra-class correlation = 0.74). A parallel-arm study powered at 0.90 would require 22 participants to detect an absolute change in RHI of 0.40 units (equal to ∼25% change in this sample), whereas a crossover study would require 12 participants. In conclusion, we have demonstrated that PAT can be used to assess endothelial dysfunction in adults with the metabolic syndrome as reliably as in healthy samples.

## Introduction

Over the last two decades, the critical role of endothelial dysfunction in atherosclerosis has been documented in *in vitro* studies, animal models, longitudinal studies and intervention trials.^1^ Translation of this knowledge to clinical settings has been limited by the fact that tests of endothelial function are either very invasive (angiography) or difficult to standardize for clinical use [flow-mediated dilation (FMD) via ultrasound].^2^ Peripheral arterial tonometry (PAT) has been proposed as an alternative method for assessing endothelial dysfunction.^3^ The PAT test is noninvasive, less operator-dependent and potentially less expensive to perform. Similar to a brachial artery FMD assessment, change in blood flow is measured before and during reactive hyperaemia induced by forearm ischaemia. A low reactive hyperaemia index (RHI) has been shown to predict coronary endothelial dysfunction with 80% sensitivity and 85% specificity in a clinical setting.^4^ Low scores also independently predicted adverse cardiovascular events over a 7-year follow-up.^5^

Before PAT can be widely adopted for clinical risk assessment, it is important to understand day-to-day variations. While there have been several reports of robust test–retest reliability of PAT, these studies have been conducted in healthy individuals or in patients with established disease.^6–10^ Given the increased risk of diabetes and cardiovascular disease (CVD) in adults with the metabolic syndrome,^11,12^ it is important to verify the reliability of PAT and to provide population-specific power and sample size estimates that can guide clinical trial design. Additionally, given that a reduced RHI has been associated with hyperglycaemia,^13,14^ it is useful to investigate whether daily variations in glucose and insulin influence PAT reliability. Therefore, the purpose of this study was to assess PAT test–retest reliability in adults with the metabolic syndrome and to use variability metrics to provide sample size and power estimates for a range of study designs. Secondary purposes were to examine the correlation between PAT scores and fasting glucose and insulin and to determine whether PAT variability was associated with fluctuations in fasting glucose and insulin.

## Methods

### Participants

These data were collected as part of a study on postpran-dial glycaemia in individuals with the metabolic syndrome.^15^ Men and women aged 40–65 years with a body mass index (BMI) >30 kg/m^2^ were recruited for the study through local advertisements and the clinic volunteer roster. All participants were required to meet the criteria for the metabolic syndrome as defined by the National Cholesterol Education Program (Adult Treatment Panel III),^16^ be in otherwise good health and not be taking any medications known to affect glucose metabolism. A total of 41 individuals were screened for the study, of which 18 failed the screening criteria and 3 withdrew from the study prior to randomization. A total of 20 participants completed the full protocol, and their characteristics at screening are reported in Table 1.

### Protocol

As part of the postprandial study, participants underwent testing on five occasions, each separated by a minimum 1-week period. All tests were performed in the morning after a 12-h fast. Endothelial function and arterial stiffness were assessed via PAT (EndoPAT; Itamar Medical Ltd, Caesarea, Israel). Vascular tests were performed in a sitting position in a quiet, dimly-lit, temperature-controlled room (70°F–75°F). Thimble-shaped pneumatic probes were applied to the index fingers of each hand, and an occlusion cuff connected to a rapid cuff inflator (Hokanson, Bellevue, WA, USA) was applied to one forearm. Following a 10-min rest period, PAT signals were recorded continuously during a 5-min baseline period, a 5-min occlusion period and a 5-min post-deflation period. During the occlusion period, the cuff was rapidly inflated to 250 mmHg to induce ischaemia in one arm.

At the conclusion of each test, proprietary EndoPAT software calculated two indices that approximated endothelial dysfunction and arterial stiffness. The RHI was calculated as follows: the ratio of the occluded arm's mean pulse wave amplitude at 90–150 s post-deflation to the mean amplitude of the same arm at baseline divided by the same ratio from the control arm, the quotient of which is multiplied by a proprietary baseline correction factor (Itamar Medical Ltd). An alternative method of calculating RHI, designed by Framingham Heart Study researchers [Framingham reactive hyperaemia index (fRHI)], was also utilized. The fRHI analyses the data from 90–120 s post-deflation, does not include the baseline correction factor and applies a natural logarithmic transformation to the final ratio.^13^ Evidence from the Framingham Heart Study suggests that there is a stronger correlation between fRHI and cardiovascular risk than RHI.^13^ For both RHI and fRHI, lower scores indicate greater endothelial dysfunction.

Arterial stiffness was approximated by the augmentation index (AI), which is calculated from the pulse waveform collected during the baseline period, through software identification of the systolic peak (P_1_) and reflected wave (P_2_) inflection points. The difference between these peaks, presented as a percentage of the peak wave [AI = (P_1_ − P_2_)/P_1_ × 100], represents the degree to which arterial stiffness increases central systolic blood pressure. Since heart rate can significantly influence the pulse waveform,^17^ an alternative way of presenting AI is to standardize it to a heart rate of 75 bpm (AI@75). For both AI and AI@75, higher scores indicate greater arterial stiffness.

Fasting blood samples were obtained by finger prick and intravenous cannulization (BD Blunt Plastic Cannula, Mississauga, ON, Canada). Glucose analysis was performed using a YSI model 2300 STAT analyser (YSI Inc., Yellow Springs, OH, USA), and insulin levels were measured using enzyme immunoassay kits (ALPCO Diagnostics, Salem, NH, USA and EMD Millipore Corporation, Billerica, MA, USA).

The study protocol was approved by the Western Institutional Review Board (Seattle, WA, USA), and written informed consent was obtained from all participants prior to starting the study. All tests were completed at Glycemic Index Laboratories Inc., Toronto, ON, Canada.

### Statistical analyses

All analyses were conducted using Statistical Analysis Software (SAS v9.2; SAS Institute Inc., Cary, NC, USA). Variables were tested for normality, and a natural log transformation was applied where appropriate. Variability in PAT metrics and metabolic parameters across the five visits were investigated using the mixed model procedure, with participant treated as a random factor and visit treated as a fixed effect. Test–retest reliability was primarily assessed with the intra-class correlation (ICC), calculated as $S_{b}^{2}-S_{w}^{2}/S_{b}^{2}+S_{w}^{2}$, where ‘ $S_{b}^{2}$’ represents the between-subject variance and ‘ $S_{w}^{2}$’ represents within-subject variance.^18^ As in previous studies, the ICC was computed using raw, non-normalized data for each individual, and a mean ICC is reported for each variable.^7^ A second measure of variability, the coefficient of variation (CV), was appropriate for RHI given its non-negative values.^19^ CV was calculated as [standard deviation (SD)/mean] × 100. Correlation and paired mean differences were also used to examine stability of each measurement over time. We examined correlations among RHI, fRHI, AI and AI@75 and day-to-day fluctuations in fasting glucose and insulin. Finally, we performed power calculations to produce sample sizes for crossover and parallel-arm designs. The tables and figures depict mean ± standard error of the mean (SEM) unless otherwise noted.

## Results

**Figure 1:**
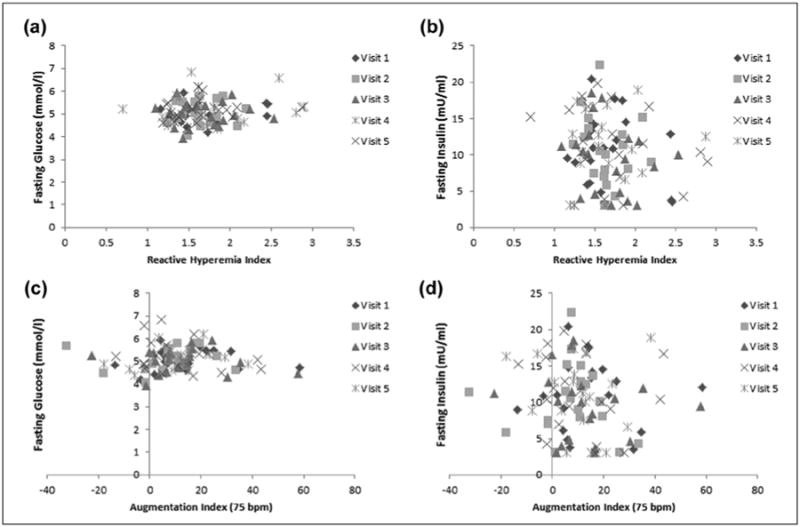
Relationship between peripheral arterial tonometry–derived measures of (a and b) endothelial dysfunction (reactive hyperaemia index) and (c and d) arterial stiffness (augmentation index standardized for heart rate of 75 bpm) with (a and c) fasting glucose and (b and d) insulin levels. All correlations were statistically non-significant.

Daily and overall means of PAT parameters, glucose and insulin are displayed in Table 2. As expected, correlations between paired measurements (i.e. visit 1 vs visit 2; visit 2 vs visit 3) were statistically significant (*p* < 0.05) and moderate to high in strength (‘r’ ranging from 0.22 to 0.87). The CV for RHI was 23.6%, and the ICC for both RHI and fRHI was 0.74. The ICC for AI and AI@75 were 0.88 and 0.86, respectively. The primary measure of variability was the average of the absolute difference between each pair of visits and is shown in Table 2. Variability in PAT was not associated with variability in glucose or insulin, and PAT results were not associated with fasting levels of glucose or insulin across the five visits (Figure 1).

Table 3 contains sample size calculations for both parallel-arm and crossover study designs for RHI, fRHI and AI@75 for varying magnitudes of treatment effects. For all sample sizes, α = 0.05 and power set at 0.80 or 0.90. For example, a parallel-arm study powered at 0.90 would require 22 participants to detect an absolute change in RHI of 0.40 units (equal to ∼25% change in our sample), whereas a crossover study would require only 12 participants. For AI@75, a parallel-arm study powered at 0.90 would require 284 participants to detect an absolute change in RHI of 4.0 units (equal to ∼50% change in our sample), whereas a crossover study would require 85 participants.

## Discussion

In this study of adults with the metabolic syndrome, we have demonstrated excellent test–retest reliability of PAT-derived measures of endothelial function and arterial stiffness. Our results are similar or more robust to those reported in previous studies of healthy participants,^6–9^ coronary artery disease patients^10^ and the general population.^14^ In contrast to large epidemiological studies that found positive associations between RHI and hyperglycaemia,^13,14^ we observed no relationship between RHI or fRHI and fasting glucose or insulin levels.

Brachial FMD has been used in research settings for decades to measure endothelial dysfunction. As summarized in a recent review,^2^ this method has been useful in estimating cardiovascular risk and evaluating interventions, but the associated costs, operator-dependence and susceptibility to external factors have limited its use in large-scale trials and for clinical decision-making. Additionally, FMD is technically challenging to complete and laboratories have individual protocols for performance and standardization. Automated edge-detection software and published methodological guidelines are helpful, but substantial variability still exists. Digital PAT testing presents a promising alternative to FMD by providing a similar assessment with a lower cost, an operator-independent procedure and, as we have shown, little day-to-day variability.

As an emerging technique, the clinical significance and prognostic value of PAT are still being established. Similar to FMD, the RHI has been shown to correlate with coronary artery vasodilation^4^ and be nitric oxide dependent.^20^ In both the Framingham Heart Study and the Gutenberg Heart Study, the RHI was associated with traditional cardiovascular risk factors such as obesity, dyslipidemia, hypertension, diabetes mellitus and smoking.^13,14^ Low RHI has been observed in individuals with established CVD and those at high risk compared to individuals with low to moderate risk.^21,22^ Importantly, an RHI below 1.49 has been associated with a higher rate of adverse cardiovascular events over a 7-year period.^5^ However, studies comparing RHI and FMD indicate that they are measuring distinct components of vascular health. Three studies reported positive correlations between FMD and RHI (r = 0.31–0.55),^23–25^ and three studies reported no correlation.^13,14,26^ Given that FMD and RHI are both associated with CVD but do not appear to be equivocal assessments, it is plausible that assessing endothelial dysfunction in different vascular beds could substantially contribute to risk stratification.

In contrast to the PAT-derived measures of endothelial dysfunction, the validity and clinical significance of PAT-derived measures of arterial stiffness have yet to be determined. Arterial stiffness is traditionally assessed via carotid-femoral pulse wave velocity (PWV) or radial applanation tonometry,^27–29^ and both of these methods have demonstrated that greater arterial stiffness is independently predictive of cardiovascular morbidity and mortality.^30–33^ To our knowledge, no studies have validated PAT with PWV, while two have compared it to radial applanation tonometry with promising results (correlations of 0.68–0.88).^34,35^ While the PAT device automatically provides both RHI and AI for each test, neither the large epidemiological studies that have examined the association between CVD and RHI nor the clinical trials that demonstrated the prognostic significance of RHI present any data for AI.^5,13,14^ Therefore, the clinical significance of PAT-derived arterial stiffness is unknown. This study has shown that AI has a high degree of test–retest reliability, with an ICC of 0.86, but more work is needed to elucidate the value of this measurement.

Large population-based studies of vascular health have suggested that hyperglycaemia is related to both endothelial dysfunction and arterial stiffness.^13,36^ Greater glycaemic variability, measured via continuous subcutaneous glucose monitoring, is also associated with endothelial dysfunction in adults with normoglycaemia, hyperglycaemia and Type 2 diabetes.^37^ Our laboratory has demonstrated that variability in both fasting glucose and insulin is directly associated with variability in FMD in Type 2 diabetes.^38^ In this study, we did not find any relationship between PAT-derived measures of endothelial dysfunction and arterial stiffness with variability of fasting glucose and insulin. This may be due to the relatively narrow range of fasting glucose (3.94–6.84 mmol/L) and insulin (3.0–22.32 mU/mL) levels that we observed, which are below the diagnostic threshold for type 2 diabetes. Notably, in our previous study of PAT reliability in young healthy adults,^7^ day-to-day change in fasting glucose levels was not significantly correlated with day-to-day change in RHI. Sample size must be considered here, as larger epidemiological studies have sufficient power to detect moderate correlations. Additionally, the pathophysiology of vascular health is complex and influenced by a host of factors.^2^ Endothelial cells in separate vascular beds have differing structural and metabolic components, which could influence how they are affected by hyperglycaemia.^39^ It is possible that PAT is not as susceptible to variations in glycaemia as FMD and carotid-femoral PWV or that small elevations in glucose and insulin do not affect vascular health to the clinically evident degree that substantial (i.e. diabetes-level) elevations do. Nevertheless, researchers are encouraged to consider assessing and controlling for hyperglycaemia in studies of vascular health.

## Study limitations

Our sample size of 20 adults is relatively small, though comparable to previous studies of PAT reliability.^6,7,10,40^ The five testing sessions occurred during a 5-week period, and therefore, we cannot draw conclusions about PAT reliability over longer periods of time. We did not collect data on other biological or physiological parameters previously found to be related to vascular health (such as lipids, lipoproteins and blood pressure) and cannot comment on how these variables may influence daily variation in RHI or AI. In addition, we did not collect data on medical therapy unrelated to the primary outcome of the larger clinical trial (glucose metabolism), and participants may have been taking medications known to affect vascular health (e.g. statins and anti-hypertensive medications). However, we believe that the crossover design of the study, which controls for individual differences to a greater degree than parallel-arm studies, attenuates any effect that other medications may have had on the results. A key strength of our study is the metabolic syndrome sample, as no previous studies have examined PAT reliability in this population, and the five repeated measurements, whereas previous studies have included only two repeated measurements. By considering the within-subject variability that we observed, researchers will be better equipped to design adequately powered clinical trials to assess the effect of interventions on digital PAT.

## Conclusion

Endothelial dysfunction and arterial stiffness are important components of vascular health that can be used to classify CVD risk and evaluate interventions. PAT testing is a novel approach with promising potential to provide reliable and clinically meaningful assessment of vascular health. While more research is needed to fully elucidate the clinical and prognostic significance of PAT, the current evidence indicates that PAT is a useful technique that can be incorporated relatively easily into research and clinical laboratories. In this study, we have demonstrated that PAT can be used to assess endothelial dysfunction and arterial stiffness in adults with the metabolic syndrome as reliably as in healthy samples.

## Figures and Tables

**Table 1:** Participant characteristics at study enrolment (n = 20).

	Mean ± SE
Female (%)	60
Age (years)	54.0 ± 1.8
Body mass index (kg/m^2^)	37.5 ± 1.8
Waist circumference (cm)	110.2 ± 1.4
Glucose (mmol/L)	5.1 ± 0.1
HDL cholesterol (mmol/L)	1.2 ± 0.1
Triglycerides (mmol/L)	2.2 ± 0.3
Systolic blood pressure (mmHg)	128.5 ± 4.4
Diastolic blood pressure (mmHg)	79.3 ± 3.0

SE: standard error; HDL: high-density lipoprotein.

**Table 2:** Fasting peripheral arterial tonometry and metabolic parameters (n = 20).

	Visit 1	Visit 2	Visit 3	Visit 4	Visit 5	Grand mean	Mean variability^a^
RHI	1.7 ± 0.1	1.6 ± 0.1	1.7 ± 0.1	1.7 ± 0.1	1.6 ± 0.1	1.7 ± 0.0	0.3 ± 0.0
fRHI	0.3 ± 0.1	0.2 ± 0.0	0.3 ± 0.1	0.2 ± 0.1	0.2 ± 0.1	0.2 ± 0.0	0.2 ± 0.0
AI	14.3 ± 3.8	8.0 ± 3.8	14.4 ± 4.0	12.8 ± 3.9	11.7 ± 3.1	12.2 ± 1.6	8.6 ± 1.1
AI@75	14.0 ± 3.5	7.6 ± 3.2	12.3 ± 3.7	11.2 ± 3.3	10.5 ± 2.8	11.1 ± 1.5	7.8 ± 1.1
Blood glucose (mmol/L)	5.1 ± 0.1	5.1 ± 0.1	5.0 ± 0.1	5.2 ± 0.2	5.2 ± 0.1	5.1 ± 0.1	0.3 ± 0.0
Serum insulin (mU/mL)	11.1 ± 1.1	10.3 ± 1.1	9.6 ± 1.1	11.4 ± 1.2	10.7 ± 1.2	10.6 ± 0.5	4.0 ± 0.4

RHI: reactive hyperaemia index; fRHI: Framingham reactive hyperaemia index; AI: augmentation index; AI@75: augmentation index standardized for heart rate of 75 bpm.

Data are represented as mean ± standard error.

a Calculated as average of the absolute value of the difference in each measure between each pair of visits (e.g. |visit 1 – visit 2|, |visit 1 – visit 3|, |visit 2 – visit 3|).

**Table 3:** Sample sizes required to detect significant treatment effects in PAT variables in parallel-arm and crossover study designs.

RHI					fRHI					AI@75				
		
Magnitude of effect	Parallel arm^a^		Crossover^b^		Magnitude of effect	Parallel arm^c^		Crossover^d^		Magnitude of effect	Parallel arm^e^		Crossover^f^	
	Power					Power					Power			
	0.80	0.90	0.80	0.90		0.80	0.90	0.80	0.90		0.80	0.90	0.80	0.90
0.20	63	83	32	42	0.10	115	158	59	78	4.0	212	284	64	85
0.40	17	22	10	12	0.20	31	41	17	21	8.0	54	72	18	23
0.60	8	11	6	7	0.30	14	19	9	11	12.0	25	33	9	12
0.80	5	7	5	5	0.40	9	11	6	7	16.0	15	19	7	8
1.00	4	5	4	4	0.50	6	8	5	6	20.0	10	13	5	6

PAT: peripheral arterial tonometry; RHI: reactive hyperaemia index; fRHI: Framingham reactive hyperaemia index; AI@75: augmentation index standardized for heart rate of 75 bpm; SD: standard deviation.

a Mean = 1.67, SD = 0.39.

b Mean = 1.67, SD = √2 × SD_within_ = 0.39.

c Mean = 0.24, SD = 0.27.

d Mean = 0.24, SD = √2 × SD_within_ = 0.27.

e Mean = 11.14, SD = 14.66.

f Mean = 11.14, SD = √2 × SD_within_ = 11.21.
